# Transcriptional Factors Related to Cellular Kinetics, Apoptosis, and Tumorigenicity in Equine Adipose-Derived Mesenchymal Stem Cells (ASCs) Are Influenced by the Age of the Donors

**DOI:** 10.3390/ani15131910

**Published:** 2025-06-28

**Authors:** Ekaterina Vachkova, Stefan Arnhold, Valeria Petrova, Manuela Heimann, Tsvetoslav Koynarski, Galina Simeonova, Paskal Piperkov

**Affiliations:** 1 Department of Pharmacology, Animal Physiology, Biochemistry and Chemistry, Faculty of Veterinary Medicine, Trakia University, 6000 Stara Zagora, Bulgaria; valeria.petrova1075@gmail.com; 2 Institute of Veterinary Anatomy, Histology and Embryology, Justus-Liebig-University of Giessen, 35392 Giessen, Germany; stefan.arnhold@vetmed.uni-giessen.de (S.A.);; 3 Department of General Animal Husbandry, Faculty of Veterinary Medicine, Trakia University, 6000 Stara Zagora, Bulgaria; tkoynarski@gmail.com; 4 Department of Veterinary Surgery, Faculty of Veterinary Medicine, Trakia University, 6000 Stara Zagora, Bulgaria; 5 Department of Mathematical Analysis and Applications, Faculty of Mathematics and Informatics, University of Veliko Tarnovo, 5003 Veliko Tarnovo, Bulgaria; ppiperkov@yahoo.com

**Keywords:** equine ASCs, aging, tumorigenic and apoptotic transcriptional factors, cellular kinetic

## Abstract

The impact of donor age on Adipose-derived mesenchymal stem cell (ASC) functionality and safety remains insufficiently characterized, particularly in equine models. This study investigates the influence of age on ASCs proliferation dynamics and the expression of tumorigenic and apoptosis-related markers. Equine ASCs were isolated from juvenile (<5 years), middle-aged (5–15 years), and geriatric (>15 years) horses and assayed across multiple passages. The relative mRNA expressions of pluripotency (Oct4), tumorigenic (CA9), and apoptosis-related (Bax and Bcl 2) markers were evaluated. The Gompertz growth model, population doubling time (PDT), and tissue non-specific ALP activity also followed. The expression of pluripotency and tumorigenic markers showed passage-dependent up-regulation, raising concerns about prolonged culture expansion. Apoptotic regulation displayed a shift with aging, as evidenced by alterations in the Bax/Bcl2 ratio, suggesting compromised cell survival in older ASCs. An age-associated decline in proliferation rates was established, as evidenced by declining alkaline phosphatase (ALP) activity. These findings underscore the necessity for stringent age-based selection criteria in equine stem cell therapies and the challenges associated with using autologous stem cells for regenerative therapies in aged horses. Future research should focus on molecular interventions to mitigate age-related functional decline, ensuring the safety and efficacy of ASCs-based regenerative medicine in equine practice.

## Simple Summary

Donors’ aging affects stem cells’ ability to regenerate damaged tissues, which is crucial for treating injuries in horses. This study examined how the age of donor horses influences the growth, survival, and potential tumor risks of adipose-derived mesenchymal stem cells (ASCs). Cells from young (<5 years old), middle-aged (5–15 years old), and elderly (>15 years old) horses were analyzed to assess their ability to multiply, maintain function, and avoid harmful genetic changes over time. The proposed Gompertzian model illustrates the proliferative ability of cells and, together with the Bax/Bcl2 ratio, could be a good predictive algorithm concerning the functional state of the isolated cells for detecting potential senescence. The findings show that ASCs from older horses have slower growth rates, a reduced ability to survive, and an altered expression of genes linked to tumor formation and apoptotic processes. These age-related changes raise concerns about the effectiveness and safety of using autologous horse stem cells for treatments later in life. However, the study also highlights potential ways to improve stem cell therapies by identifying factors influencing cell aging. This research provides valuable insights for veterinarians and scientists working on regenerative medicine, helping to develop safer and more effective treatments for equine injuries and age-related conditions.

## 1. Introduction

The equine superficial digital flexor tendon (SDFT) is a crucial energy-storing structure that frequently suffers injury, particularly in performance horses engaged in high-impact activities such as galloping and jumping [1]. The recovery process varies based on age, general health, and injury severity [2]. Geriatric horses (>15 years old) are at a heightened risk of SDFT injuries due to increased locomotor activity when housed in open barns or large groups [3,4]. Aging tendons undergo structural changes, primarily driven by post-translational collagen modifications, particularly glycation [5]. These modifications alter the biomechanical properties of type I collagen, making tendons more susceptible to strain-induced injuries [1]. While healing may take 1–2 years, complete biomechanical restoration rarely occurs, predisposing the tendon to recurrent trauma [6].

Although stem cells and their products are often used in equine medicine to treat the aforementioned disorders, they can also be used in ophthalmology, wound healing, equine metabolic syndrome, and obstetric diseases [7,8,9,10]. Studying their properties is important in allogeneic, autologous, and heterologous transplantation since there is data on the application of stem cells, even from horses to dogs [11]. Mesenchymal stem cells (MSCs) and their extracellular vesicles (EVs) have shown significant promise in regenerative medicine, particularly for tendon repair and regeneration [12]. Among MSC sources, adipose-derived mesenchymal stem cells (ASCs) are attractive due to their abundance, accessibility, and multipotency. Enzymatic digestion of adipose tissue using collagenase yields stromal vascular fraction (SVF), which consists of a heterogeneous mixture of cell types, including blood-derived cells, pericytes, endothelial progenitors, and adherent stromal cells [13]. The non-adherent hematopoietic cells are eliminated upon culture expansion, leaving a purified ASC population [14,15].

Cultural conditions can significantly influence the MSC phenotype, and long-term in vitro culture may induce spontaneous mutations leading to malignant transformation [16]. In human medicine, tumorigenic cell impurities, particularly residual pluripotent stem cells (PSCs), are a significant safety concern in stem cell-based therapies [17,18] since they can be a significant risk factor for tumorigenesis when the products contain them [19]. Although some of the criteria for pluripotency include tri-linear differentiation in vitro and teratoma formation in vivo, the PSCs identified through markers, such as stage-specific embryonic antigen-4 (SSEA4), tumor rejection antigens (TRA-1-60 and TRA-1-81), alkaline phosphatase (ALP), Oct4, and Nanog, pose a potential oncogenic risk [20,21]. Tissue-nonspecific alkaline phosphatase shows high expression in pluripotent cells such as embryonic germ and stem cells and induced pluripotent stem cells (iPSCs) [17,22,23,24]. Additionally, the expressions of apoptotic and anti-apoptotic markers, including Bcl-2-associated X protein (Bax) and B-cell lymphoma 2 (Bcl-2), serve as prognostic indicators in cancer biology [25,26,27] and may provide insights into the tumorigenic potential of ASCs.

Although ASCs hold great therapeutic promise, emerging evidence suggests their potential role in tumor progression. ASCs have been shown to enhance breast cancer cells’ proliferation and metastatic capacity, accelerate tumor growth in co-culture models, and increase interleukin-6 secretion, which activates oncogenic signaling pathways [28]. Furthermore, the secretion of EVs, including exosomes and microvesicles, by ASCs contributes to tissue regeneration and vascularization [29]. Physiologically, they regulate biological functions, homeostasis, and the body’s immune response. However, these EVs may also facilitate tumorigenic processes by delivering growth factors and interleukins that promote angiogenesis, immunosuppression, the mesenchymal-to-epithelial transition, and chemotherapy resistance [30].

Despite the widespread investigation of ASCs in human medicine, limited research has explored their tumorigenic potential in equine models. The donor’s age, differentiation capacity, inflammatory responses, and paracrine effects must be carefully evaluated in stem cell-based therapies for horses.

To establish safety criteria for clinical applications of equine ASCs, depending on the donors’ age and passage number, this study aims to do the following:

- Assess cellular kinetics and evaluate the best-fitting growth pattern of the isolated cells by comparing three mathematical models for population growth—exponential, logistic, and Gomperzian;
- Assess apoptotic stage by estimating the Bax/Bcl2 ratio;
- Assess pluripotency and tumorigenic markers in equine ASCs by evaluating the relative mRNA expression of Oct 4, CA9, and tissue non-specific ALP activity.

## 2. Materials and Methods

### 2.1. Cell Isolation

Subcutaneous adipose tissue samples were collected from 12 mixed-breed horses across three age groups: <5 years old (juvenile), 5–15 years old (adult/middle-aged), and >15 years old (elderly). Animals were operated on under neuroleptanalgesia using intravenously administered detomidine hydrochloride—0.01 mg/kg (Domosedan^®^, Orion Pharma, Espoo, Finland) and butorphanol tartrate—0.025 mg/kg (Butomidor^®^, Richter Pharma, Wels, Austria), supplemented by local infiltration with 2% lidocaine. The surgical site was aseptically prepared, followed by a longitudinal incision (~5–6 cm) in the left or right gluteal region (Regio gluteal sinistra) to extract 2 cm^3^ of adipose tissue. Tissue samples were mechanically minced and enzymatically digested with collagenase for two hours.

Peripheral blood samples from six animals were collected in anticoagulant tubes and centrifuged (3000 RPM, 10 min, room temperature) to isolate the buffy coat. Leukocytes were used as positive and negative controls for specific MSC markers. The experimental design adhered to national animal welfare regulations and was approved by the National Ethics Commission (Permit No. 322/09.12.2021, Bulgarian Food Safety Agency, Bulgarian Ministry of Agriculture).

### 2.2. ASCs’ Tri-Lineage Differentiation

At the fifth passage, pooled ASCs from each age group were seeded in a basal medium (DMEM-Dulbecco’s Modified Eagle’s Medium, 10% FBS-fetal bovine serum, and 10 mL/L of mixed antibiotic/antimycotic solution) and maintained in a humidified, 5% CO_2_ environment at 37 °C. The cells were seeded in 12-well plates and, after reaching confluency, were left for an additional 24 h (growth arrest). Tri-lineage differentiation was induced as follows:

- Adipogenesis: Cells were cultured in adipogenic medium (DMEM-HG, HS-heat-inactivated horse serum, dexamethasone, insulin, indomethacin, IBMX, and antibiotic/antimycotic solution) for 18 days [31]. Intracellular lipid droplet (LD) formation was confirmed using Oil-Red-O staining.
- Osteogenesis: Cells were incubated in osteogenic medium (DMEM, FBS, ITS, dexamethasone, β-glycerol phosphate, ascorbic acid, glutamine, antibiotic/antimycotic solution). Calcium deposits were visualized on day 21 via Alizarin Red S staining.
- Chondrogenesis: Cells were cultured in a chondrogenic medium (ITS, FBS, dexamethasone, TGFβ1, L-ascorbic acid, glutamine, antibiotics) and maintained for 21 days in 12-well plates. Proteoglycan deposition was confirmed with Alcian Blue staining.
- Negative controls: Negative controls were cultured in parallel using a basal medium. All micrographs were taken with a Leica DM1000 LED inverted microscope (Zurich, Switzerland) equipped with a DMi1 camera, 5.0-megapixel resolution, and a Leica Application Suite Core software version 3.4.0 platform.
- Equine lipoma cells: The equine lipoma cells were obtained as described by Arnhold et al., 2019 [32], and used as a positive control for apoptotic and tumorigenic markers.

### 2.3. qPCR Analysis

Total RNA was extracted using the Universal RNA Purification Kit (EURx, Gdańsk, Poland). The concentration and quality of the samples were determined spectrophotometrically with an Agilent Cary 60 UV/Vis spectrophotometer. To ensure high-quality template preparation, all RNA samples were purified to eliminate potential genomic DNA contamination using an RNAse-Free DNase Set (QIAGEN, Hilden, Germany). cDNA synthesis was performed using the RevertAid First Strand cDNA Synthesis Kit (Thermo Scientific, Waltham, MA, USA). Gene expression was quantified via the KAPA SYBR FAST Master Mix Kit (Roche, Basel, Switzerland) using a Gentier 96E thermocycler (Xi’an Tianlong Science and Technology, Xi’an, China). Four candidate housekeeping genes (HKs) were tested (GAPDH, 18S, B2M, HPRT), and the most stable combination (GAPDH and B2M) was selected via NormFinder [33]. Primer sequences for CD markers, tumorigenic markers, and housekeepers are listed in (Table S1). The primers were designed using web-based software algorithms such as Primer Blast NCBI (www.ncbi.nlm.nih.gov) based on Primer3web version 4.1.0 and Oligo Explorer v. 1.4 Beta (https://www.genelink.com/tools/gl-oe.asp) accessed in period October- December 2022. The qPCR row data were normalized as described by Vandesompele et al., 2002 [34] and calculated relative to the geometric mean of both HKs for each replicate.

### 2.4. Growth Kinetics

ASCs from passages 3–5 were seeded in a 24-well plate at an initial concentration of 0.5 × 10^4^ cells/well and cultured in a basal medium (DMEM, 10% FBS, and antibiotic–antimycotic mixture) in humidified 5% CO_2_ conditions. Cell growth was monitored at 24 h intervals over 192 h using a Scepter™ 3.0 cell counter (MerckMillipore™, Darmstadt, Germany).

Three possible laws for population growth—exponential, logistic, and Gomperzian—were presented as mathematical models and tested to evaluate the best-fitting growth pattern.

The measurements were made at equal intervals at points $t_{0},\ldots ,t_{n}$. The values of the measurements are denoted by $y_{0},\ldots ,y_{n}$. In this case, n=8, and the sample consists of 9 numbers. For convenience, $t_{i}=i$ for i=0,1,…,n, i.e., time is measured in days.

- Exponential law. The equation is

(1) $$y=exponent(t)=y_{0}\cdot C^{t}$$

where $y_{0}$ is the value of the function when t=0.

- Logistic law. It corresponds to the differential equation [35]

(2) $$\frac{dy}{dt}=r\cdot y-\frac{r\cdot y^{2}}{K}$$

where K is the capacity (the maximum theoretical value the population can expand), and r is the growth rate. In explicit form, the logistic function has the equation

(3) $$y=logistic(t)=\frac{K}{\frac{K-y_{0}}{y_{0}}\cdot e^{-r\cdot t}+1}$$

- Gompertzian law. It corresponds to the differential equation

(4) $$\frac{dy}{dt}=a\cdot y\cdot ln\frac{K}{N}$$

where K is the capacity and a is a specific coefficient of the growth rate. The equation in explicit form is

(5) $$y=Gompertz(t)=K\cdot {\left(\frac{y_{0}}{K}\right)}^{e^{-a\cdot t}}$$

For each sample (passages 3–5 and horses aged up to 5, up to 10, and over 15 years old), the best approximation was calculated with each listed function type (exponent, logistic, Gompertz). The error was estimated using the mean absolute error (MAE) method or the root mean square error (RMSE) method.

(6) $$MAE(f)=\frac{1}{9}\cdot \sum_{i=0}^{8}\left|f(t_{i})-y_{i}\right|$$

(7) $$RMSE(f)=\sqrt{\frac{1}{9}\cdot \sum_{i=0}^{8}{\left(f(t_{i})-y_{i}\right)}^{2}}$$

The error was minimized using the cloud version of Wolfram Mathematica (https://www.wolframcloud.com) accessed in period December 2024–January 2025.

Additionally, growth curves were modeled using the Gompertz equation and visualized by GraphPad Prism 10 Software LLC (Boston, MA, USA).

The following exponential model corresponding equation was applied to evaluate each passage’s population doubling time (PDT) in hours.

(8) $$PDT=\frac{Duration*log(2)}{log(FinalConcentration)-log(InitialConcentration)}$$

where “log” is the logarithm to base 2 [36].

### 2.5. ALP Activity

ALP activity was quantified in culture supernatants using a BS-120 Chemistry Analyzer (MINDRAY, Shenzhen, China) and an ALP reagent (Biolabo, Maizy, France), based on the spectrophotometrically measured p-nitrophenol formation, proportional to the ALP activity.

### 2.6. Statistical Analysis

The mean values and their standard error (mean ± SEM) of the variables were determined. The statistical significance of the differences in the values (*n* = 3–6) of the various parameters between the passages was determined using nonparametric the Mann–Whitney Test of Statistica v7.0 (StatSoft Inc., 2004, Tulsa, OK, USA). Concerning the expression of CD markers, statistical analysis was applied to positive markers to reveal the influence of donors’ age and passage number on their expression in ASCs. The negative CD markers were used to illustrate that the isolated cells meet mesenchymal stem cell identification requirements. The Spearman correlation coefficient R (significance level *p* < 0.05) of Statistica v7.0 (StatSoft Inc., 2004, Tulsa, OK, USA) was used to determine the functional relationship between the expression of observed factors.

## 3. Results

### 3.1. ASCs Tri-Lineage Differentiation

**Figure 1:**
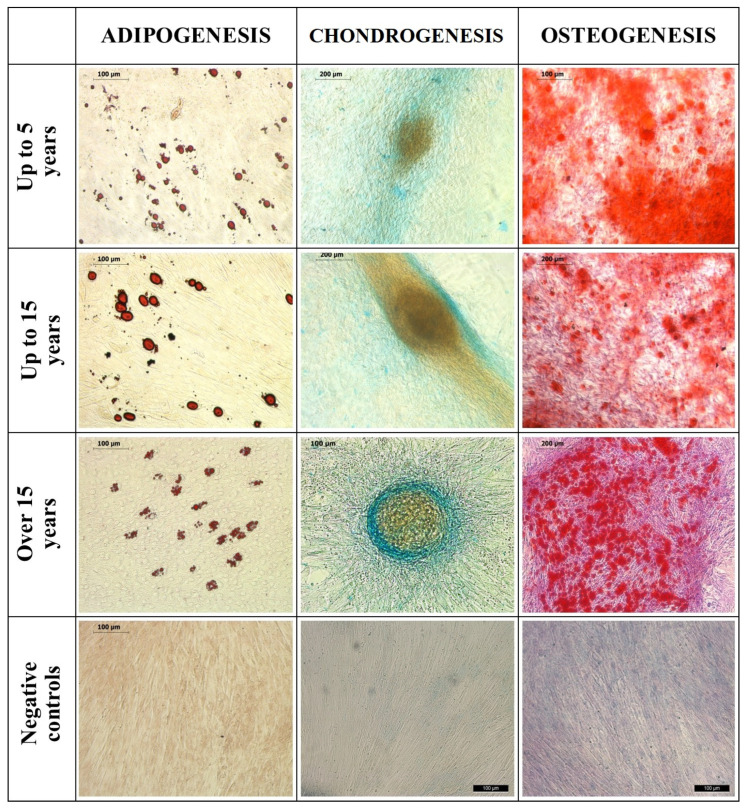
Tri-lineage differentiation of equine subcutaneous ASCs: 1st column—the samples stained with Oil Red O for adipogenesis and their negative controls; 2nd column—the samples stained with Alcian blue for chondrogenesis; 3rd column—the samples stained with Alizarin Red for osteogenesis and their negative controls. Bars are shown in micrographs.

In the fifth passage, the equine adipose-derived stem cells (ASCs) from each age group demonstrated successful tri-lineage differentiation (Figure 1).

Upon adipogenic induction, lipid droplets formed within the cells, which exhibited a strong affinity for Oil-Red-O staining, turning red. Chondrogenic induction led to the formation of micromass, and the extracellular proteoglycans were highlighted in blue using Alcian Blue staining. Osteogenic induction resulted in extracellular calcium deposits, which were positively stained with Alizarin Red, appearing as red. Negative control samples did not undergo induction and showed no affinity for these stains.

### 3.2. qPCR and ALP Activity

#### 3.2.1. Relative mRNA Expression of CD Markers

The expression of four HKs (GAPDH, 18S, B2M, and HPRT) was analyzed, and the lowest Stability value (0.171) was found for the combination of GAPDH and B2M.

**Figure 2:**
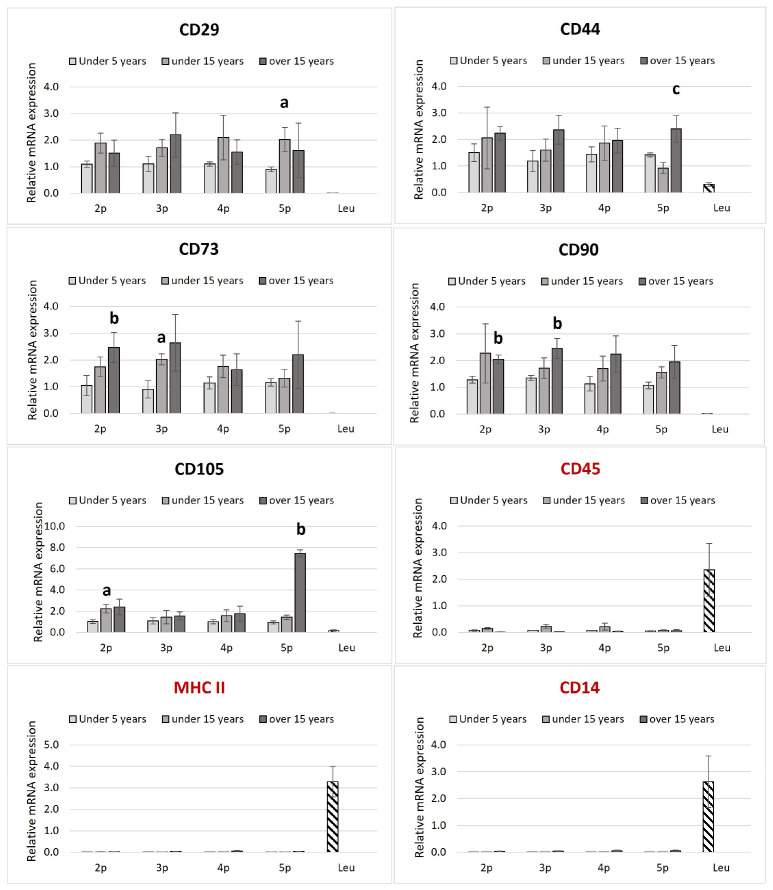
Transcriptional profile of the MSCs’ positive (in black label, CD90, CD73, CD105, CD44, and CD29) and negative (in red label, MCHII, CD45, and CD14) markers of ASCs in equines from different age groups. The relative mRNA expression is plotted in passages from two to five. The leukocytes (downward diagonal pattern) were used as a negative control for the positive ASCs markers. The age-dependent significances (*p* < 0.05) within the same passage are indicated with small letters as follows: “**a**” for differences between “up to 5 years” and “up to 15 years”; “**b**” for differences between the “up to 5 years” and “over 15 years” groups; and “**c**” for differences between “up to 15 years” and “over 15 years”. (*n* = 2–4 for ASCs and *n* = 4–6 for the leukocytes; Bars = ±SEM).

The isolated ASCs were found to express all MSC-positive CD markers tested while showing low or undetectable levels of negative markers (Figure 2).

No significant differences in positive CD markers’ expressions within passages from the same age groups were established. CD105 in the second passage, CD73 in the third passage, and CD29 in the fifth passage differed significantly between the young and middle-aged groups. When the same passages from different age groups were compared, a marked upregulation of CD 73 in the second, C90 in the second and third, and CD105 in the fifth passage was established in the elder compared to the juvenile group. CD44 was also upregulated at the fifth passage in juveniles compared to the old group.

Leukocyte samples were negative for MSC markers but positive for MHCII, CD45, and CD14.

#### 3.2.2. Relative mRNA Expression of Tumorigenic and Apoptosis-Related Markers

**Figure 3:**
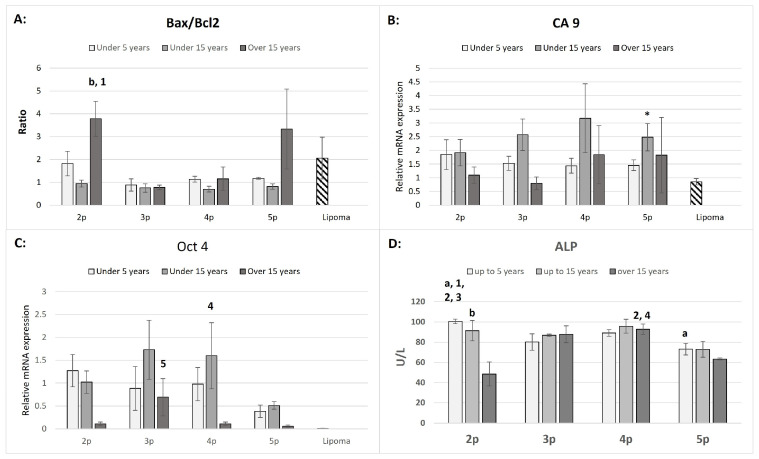
The Bax/Bcl 2 ratio (**A**), CA9 (**B**), Oct4 (**C**), relative mRNA expression in ASCs and lipoma cells (downward diagonal pattern), and ALP activity (U/L) in supernatants in ASCs (**D**) from three age groups within the passages. The age-dependent significances (*p* < 0.05) within the same passage are indicated with small letters as follows: “**a**” for differences between “up to 5 years” and “up to 15 years” old; “**b**” for differences between the “up to 15 years” and “over 15 years” groups. The passage-dependent significances (*p* < 0.05) within the same age group are indicated with numbers as follows: “**1**” for differences between 2p and 3p; “**2**” for differences between 2p and 4p; “**3**” for differences between 2p and 5p; “**4**” for differences between 4p and 5p; and “**5**” for differences between 3p and 5p. The significant difference between ASCs and lipoma cells is marked with an asterisk “*” (Bars = ±SEM, *n* = 3–5 for (**A**–**C**); Bars = ±SEM, *n* = 4–6 for (**D**)).

Bax and Bcl2 mRNA expression levels were used to calculate the Bax/Bcl2 ratio (Figure 3A).

Notably, significantly higher Bax/Bcl2 ratios were observed in the second passage of the “over 15 years” group compared to the “up to 15 years” group in the same passage and also compared to the third passage in the “over 15 years” group. Except for lipoma cells, ASCs cultures from the second passage of the “up to 5 years” group, and the second and fifth passages of the “over 15 years” group, most ratios were close to one. The mRNA expression of CA9 was significantly upregulated only in the fifth passage compared to lipoma cells. However, the variations across age groups and passages were not significant (Figure 3B). The expression of Oct 4 mRNA was notably higher in the “up to 15 years old” group at the fourth passage and the “over 15 years old” group at the third passage, while no significant expression was detected in lipoma cells (Ct values were close to the detection limit, between cycles 35–38).

### 3.3. ALP Activity

The ALP activity was significantly higher in the “up to 5 years old” group compared to other passages within the same group and relative to the different age groups (Figure 3D). This trend was consistent in the fifth passage, where ALP activity in this group remained significantly higher than in the “over 15 years old” group. The “up to 15 years old” group displayed no significant differences in ALP activity between passages, except in the second passage, where the activity was significantly higher than in the “over 15 years old” group. The highest ALP activity in the “over 15 years old” group was observed in the fourth passage, compared to the second and fifth passages in the same group. To rule out any medium-related influence, ALP activity was measured in basal medium and fetal bovine serum (FBS), which showed zero activity.

### 3.4. Correlation Analysis

All significant correlations (R) between the target genes and CD markers tested were positive (Table 1).

The strongest correlation was found between CA9 and Bcl2 in the “up to 15 years old” group. Similarly, this correlation was evident in the “up to 5 years old” group. Notably, the “up to 15 years old” group displayed the most significant functional correlations. No significant correlations were observed for lipoma cells.

### 3.5. Growth Kinetics of Equine ASCs

The exponential model deviates from approximation by both the MAE and RMSE criteria. Based on the least MAE value, the Gompertzian model best fits the cellular kinetic of equine ASCs from all age groups and passages (Table S2). RMSE values for the logistic model were slightly (0.01–0.03) lower than those for the Gompertz model in only three groups, but those variations could be assumed to be insignificant.

- “Up to 5 Years” Group

**Figure 4:**
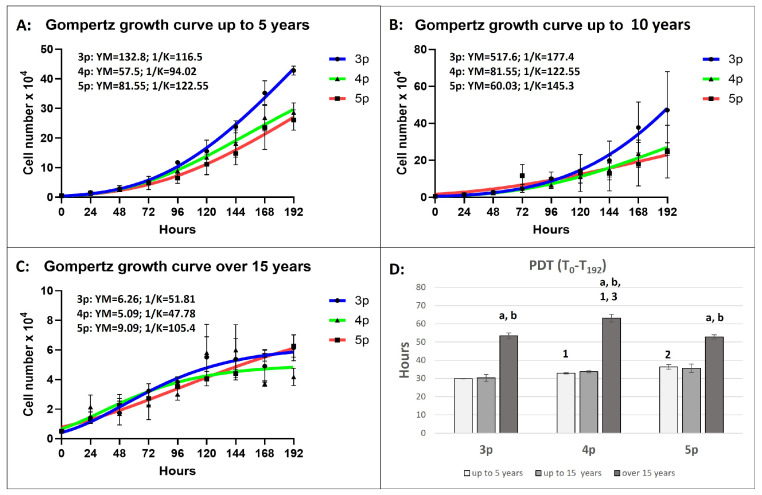
The Gompertz model growth curves follow cellular growth kinetics for a 192 h period at different passages and in different age groups (**A**–**C**), where the YM is the maximum population (cell number) at each passage, and 1/K determines the time point at which the rate of cell division is constantly increasing (the start of the exponential phase). The number of cells is plotted as a function of time in hours (Bars = ±SEM, *n* = 4). (**D**): Population doubling time (PDT) in hours. The age-dependent significances (*p* < 0.05) within the same passage are indicated with small letters as follows: “**a**” for differences between “up to 5 years” and “up to 15 years” old; “**b**” for differences between the “up to 15 years” and “over 15 years” groups. The passage-dependent significances (*p* < 0.05) within the same age group are indicated with numbers as follows: “**1**” for differences between 3p and 4p; “**2**” for differences between 3p and 5p; and “**3**” for differences between 4p and 5p (Bars = ±SEM, *n* = 4).

The growth pattern in this group followed a Gomperzian pattern across all passages, with the exponential growth phase beginning relatively late between 94.02 and 122.55 h (Figure 4A).

The cells did not enter the stationary phase. The prognostic maximum yield per passage (YM) was reached at the third passage, where the final cell number was predicted to be 132.8 × 10^4^. The population doubling time (PDT) significantly increased with each passage, with the longest PDT (36.42 h) recorded in the fifth passage.

- “Up to 15 Years” Group

Similarly to the “up to 5 years” group, the growth pattern in all passages was also Gomerzian (Figure 4B). The exponential growth phase began later, at 177 h in the third passage, where the YM value reached its peak (517.6 × 10^4^), the highest among all age groups and passages. The cells from this age group showed the highest proliferative capacity among all passages and groups. The Gompertz model revealed a lack of a stationary growth phase, and insignificant differences in PDT were observed between passages.

- “Over 15 Years” Group

The growth curve in the third and fourth passages followed a Gompertzian model, with exponential growth phases reaching 51.81 and 47.78 h, respectively (Figure 4C). The final YM value at the third passage was 20-fold and 80-fold lower than in the “up to 5 years” and “up to 15 years” groups, respectively. In the fifth passage, the final YM values were 9-fold and 6-fold lower than those in the younger groups. The PDT was significantly higher than the other two age groups, with the highest value recorded in the fourth passage (over 60 h).

## 4. Discussion

ASCs’ Tri-Lineage Differentiation and CD Marker Expression

The ability of ASCs to undergo tri-lineage differentiation into adipocytes, chondrocytes, and osteoblasts is a defining characteristic of mesenchymal stem cells (MSCs). Our results confirm that equine ASCs from all age groups retained a multipotent differentiation capacity, aligning with the minimal criteria established by the International Society for Cellular Therapy (ISCT) and the International Federation for Adipose Therapeutics and Science (IFATS) [15,37]. The expression of MSC-specific CD markers, including CD29, CD44, CD73, CD90, and CD105, further validates their identity. In contrast, the absence of hematopoietic markers (CD34, CD45, CD14) confirms the purity of the isolated ASCs [38,39]. The lack of MHC-II expression in our isolated ASCs aligns with previous findings [40] and supports their potential for allogenic applications without triggering major immune responses.

Although CD29 was robustly expressed across all samples, the expression of CD105 showed variability among different age groups, a phenomenon previously reported in equine MSC studies [41]. This inconsistency in CD105 expression could be attributed to donor variability, passage number, or species-specific differences, as suggested by De Schauwer et al., 2011 [42]. Notably, CD105 has been implicated in angiogenesis and immunomodulation, which may indicate functional differences between age groups [43].

CD90 plays a crucial role in stem cell maintenance, and its high expression in our samples is consistent with findings that link CD90 to enhanced proliferation and survival potential [41]. Similarly, CD44 has been identified as a cancer stemness marker involved in immunomodulation, particularly in bone marrow-derived MSCs [44]. The maintained expression of these markers across all age groups suggests that equine ASCs retain key mesenchymal features regardless of donor age.

Age-Related Changes in ASCs’ Function and Regenerative Potential

Our data indicate that ASCs derived from horses older than fifteen years exhibited significant molecular alterations that could compromise their regenerative potential. These findings align with studies reporting that aging reduces the proliferation, survival, and differentiation potential of MSCs across various species [45]. In particular, genes associated with stem cell homeostasis and epigenetic regulation, including DNMT1, TET-2, and TET-3, showed diminished expression in older horses, mirroring age-related declines in epigenetic plasticity observed in human MSCs [46]. Furthermore, the observed decrease in CXCR4 and Oct 3/4 expression suggests that aged ASCs may have a reduced ability to attach to injury sites and sustain long-term proliferation [47].

Alkaline Phosphatase (ALP) Activity and Growth Kinetics

The activity of ALP in the isolated ASCs in all passages within different age groups was found to be within the reference range for equines, which is 73–194 IU/L [48]. ALP activity is a widely accepted osteogenic differentiation marker and early MSC lineage commitment. Our findings demonstrate that ALP activity varied significantly across age groups, with the highest levels observed in the “up to 5 years old” group and a progressive decline with increasing age. This trend is consistent with human and bovine MSC studies, where aging is associated with reduced osteogenic potential and diminished ALP expression [49,50]. In equine ASCs, this decline in ALP activity may reflect a decreased capacity for bone regeneration in aged individuals, which has important implications for orthopedic regenerative therapies in horses [48].

The Gompertz model is a commonly used sigmoid model for analyzing growth and various datasets. It has broad applications across biology to illustrate the growth patterns of animals and plants, the development of fish, the population or size of bacteria and cancerous cells, etc. [51]. There is no available information about the previous application of the Gomperzian model for evaluating the growth kinetics in equine ASCs. Our findings revealed that the Gompertzian model best fits the ASC population growth pattern in equines, no matter the passages and donors’ ages. Based on the highest MAE and RMSE criteria in all investigated groups and passages, we conclude that the exponential model did not approximate the observed cellular kinetic pattern in equine ASCs. Regarding the established approximation to the logistic model, we should also consider the function capacity (K), where the values for the Gompertz model were notably higher in up to 15-year-old groups, and the cells showed the highest proliferative potential. The same criteria for both models did not differ dramatically in the oldest groups, where the cells showed the slowest proliferation rate. In such approximation cases, both the logistic and Gompertz models are applicable.

Growth kinetics analyses further support the notion of age-related functional decline. Younger and middle-aged ASCs followed a Gomperzian growth pattern with a shorter population doubling time (PDT) and a higher maximum yield per passage (YM), indicating a higher proliferative potential. In contrast, ASCs from horses over 15 years old displayed significantly slower proliferation, an altered growth curve, and an early transition into a stationary phase. Moreover, the nonexponential growth observed at the fifth passage in the over-15-year-old age group could be because no dividing cells were present in the population [52]. These findings align with reports that MSC proliferation declines with age due to telomere shortening, mitochondrial dysfunction, and epigenetic modifications [53,54]. The impaired proliferative capacity observed in older ASCs highlights the challenges associated with using autologous stem cells for regenerative therapies in aged horses. It underscores the need for strategies to enhance the functionality of aged ASCs, such as preconditioning with growth factors or genetic modifications.

Some authors reported a weakness in the proliferative capacity of mesenchymal stem cells from bone marrow and adipose tissue after the fourth passage in animals in the geriatric age, where increased expression of apoptotic genes, tumor suppressors, and growth factors was observed [55]. Our findings confirm such a trend, where the observed changes may indicate that aged ASCs adopt a more senescent phenotype that could impair their therapeutic efficacy in regenerative applications. Therefore, we conclude that increasing age (>5) rapidly reduces the major functions of ASCs, such as cell survival, homeostasis, and proliferation activity [45].

Correlation Between Tumorigenic Markers and ASC Age

The expression patterns of tumorigenic markers provide additional insights into the safety profile of ASCs from different age groups. CA9, a hypoxia-induced gene involved in tumor progression and metabolic adaptation, was significantly upregulated in the fifth passage compared to lipoma cells. While previous studies have linked CA9 overexpression to an increased risk of malignancy in human MSCs [56], the relatively low levels observed in early passages suggest that prolonged culture expansion rather than donor age is the primary factor driving its expression.

Similarly, Oct4 expression, a key pluripotency marker, was higher in the “up to 15 years old” group in the fourth passage and in the “over 15 years old” group in the third passage. The absence of Oct4 expression in lipoma cells suggests that its expression in ASCs is not indicative of malignant transformation but rather a transient feature of multipotency. Nonetheless, these findings emphasize the importance of passage number in stem cell characterization and the need for the strict monitoring of oncogenic markers in clinical applications.

BCL2 and BAX

The balance between pro-apoptotic and anti-apoptotic proteins within the BCL2 family is critical in regulating apoptosis, a process that is essential for cellular homeostasis, tissue remodeling, and response to environmental and metabolic stress [57]. The interplay between BCL2 and BAX, two central members of the BCL2 family, is particularly significant in the context of adipose tissue function and development. While BCL2 acts as a key anti-apoptotic protein, protecting cells by inhibiting mitochondrial outer membrane permeabilization (MOMP) and preventing cytochrome c release, BAX, a pro-apoptotic protein, promotes MOMP, triggering the apoptotic cascade [58]. The intricate balance between these two proteins is essential for maintaining cellular viability, particularly in tissues undergoing dynamic changes, such as adipose tissue, which is involved in metabolic flexibility and energy storage.

In the present study, the simultaneous upregulation of BCL2 and BAX in the youngest group of horses (3–4 years old) offers an intriguing view into regulating apoptosis in early development. The elevation of BCL2 expression in this group reflects the necessity for cellular survival during growth, as cells must persist through active growth and differentiation periods. Interestingly, the simultaneous increase in BAX expression in these younger horses indicates a compensatory mechanism that prevents excessive cell death, thereby maintaining the tissue integrity required for ongoing development. This equilibrium suggests that apoptosis is not suppressed but controlled, ensuring that it only occurs in response to specific stressors or abnormalities.

Similar findings have been reported in other species, where anti-apoptotic and pro-apoptotic gene co-expression occurs during developmental processes to balance tissue homeostasis. In murine models, for instance, the co-expression of BCL2 and BAX during kidney development supports tissue remodeling and differentiation without leading to excessive apoptosis [59]. This co-expression ensures that apoptosis is tightly regulated and only occurs when necessary to remove defective or excess cells. Similarly, in zebrafish, coordinated regulation of BCL2 and BAX during early organogenesis has been shown to prevent unchecked cell death while promoting appropriate organ development [60]. These observations underscore the importance of the BCL2/BAX ratio in regulating the balance between cell survival and death, particularly during critical developmental windows.

In adipose tissue, from where the ASCs originate, this dual regulation of BCL2 and BAX plays a crucial role in both adipogenesis and metabolic flexibility. The growth and expansion of adipose tissue during early life are highly dependent on the careful regulation of apoptosis, as this ensures that only functional adipocytes are retained while defective or excessive cells are eliminated. In the present study, the increased expression of BCL2 and BAX in younger horses may reflect a robust mechanism that facilitates the expansion of adipose tissue while ensuring that cellular turnover is controlled. This concept aligns with findings from studies on murine adipose tissue, where the balance between BCL2 and BAX ensures the proper regulation of adipogenesis and apoptosis, supporting metabolic flexibility [61].

However, as horses age, we observed a marked decline in BCL2 expression, particularly in the 15− and 15+-year-old groups. This decrease in BCL2 expression is consistent with the aging process in many mammalian tissues, where the regenerative capacity diminishes, and cells become more prone to apoptosis. Age-related changes in apoptosis regulation have been well documented, with studies showing that decreased BCL2 expression is associated with increased susceptibility to cellular damage, the loss of tissue function, and the accumulation of senescent cells [58]. As BCL2 levels decline with age, the ability of tissues to adapt to stressors and repair damage is compromised, leading to the functional decline of organs and tissues.

Interestingly, the relatively stable expression of BAX in the older age groups suggests that while apoptosis may still occur, it is not necessarily a result of dysregulated or excessive apoptotic signaling. Instead, the stable expression of BAX may reflect a basal level of apoptosis that is necessary for maintaining tissue turnover and homeostasis in aging tissues. Some studies have suggested that maintaining a minimal level of BAX expression may be required for removing damaged or dysfunctional cells, thus preventing the accumulation of cells with compromised functions, which could contribute to age-related diseases [62]. In this context, the relatively stable BAX expression observed in older horses may represent a baseline level of apoptosis that ensures the turnover of aging cells without leading to excessive tissue loss.

Age-related BCL2/BAX ratio shifts have been implicated in losing tissue resilience and regenerative potential across many species. For example, in humans, the decline in BCL2 expression and the alteration of the BCL2/BAX ratio with age has been associated with a decrease in tissue repair capacity and an increased incidence of age-related diseases such as cancer, cardiovascular disease, and neurodegenerative disorders [63]. Similarly, in murine models, aging is accompanied by a reduced ability to regulate apoptosis, which may contribute to the progressive decline in tissue function observed in older animals [64].

In our study, the observed shift in the BCL2/BAX balance, where the anti-apoptotic signaling decreases with age while the pro-apoptotic signaling remains relatively stable, may have important implications for the aging process in equine adipose tissue. This change could lead to an accumulation of senescent cells and a reduction in the ability of adipose tissue to respond to metabolic demands. This may also reflect broader changes in metabolic regulation, as aging adipose tissue becomes less responsive to endocrine signals and more prone to dysfunction.

Overall, the findings of our study emphasize the critical role of apoptosis regulation in equine ASCs during different life stages. The adaptive balance between BCL2 and BAX in younger horses underscores the importance of controlled apoptosis during tissue development and growth. However, the age-related decline in BCL2 expression observed in older horses highlights the potential consequences of disrupted apoptosis regulation, including decreased tissue resilience and metabolic dysfunction. The relatively stable expression of BAX in older horses suggests that apoptosis still plays a role in tissue maintenance, but the regulation of apoptosis becomes less dynamic with age.

These findings raise several important questions regarding the molecular mechanisms governing the BCL2/BAX balance in equine ASCs. Future studies are needed to investigate the signaling pathways that modulate the expression of these genes in response to physiological and environmental factors, including stress, diet, and metabolic disorders. Additionally, understanding how the BCL2/BAX ratio influences adipose tissue and ASCs, respectively, function in aging horses could provide valuable insights into managing age-related diseases in equines, including obesity, metabolic syndrome, and insulin resistance.

Moreover, exploring the molecular interactions between BCL2 and BAX in ASCs could lead to identifying novel therapeutic targets for enhancing tissue repair and regeneration in aging animals. This could have significant implications for improving horses’ health and longevity, particularly in equine geriatrics, where metabolic and age-related disorders are prevalent.

## 5. Conclusions

This study comprehensively analyzed the age-related molecular and functional changes in equine ASCs, highlighting their differentiation potential, proliferation dynamics, and tumorigenic marker expression. Our findings indicated that while equine ASCs retained multipotent characteristics across all age groups, aging significantly affected their regenerative capacity, growth kinetics, and apoptotic profile. The observed decline in ALP activity and proliferative potential with age suggested that younger ASCs may be more suitable for clinical applications, not only in orthopedic and tendon regeneration but also in musculoskeletal disorders, wound healing in extensive skin injuries, equine metabolic syndrome, ophthalmology, and heterologous transplantation. Additionally, the differential expression of tumorigenic markers underscored the importance of passage number in maintaining stem cell safety.

The Gompertz model best fit the equine ASCs’ kinetics, illustrating their functional condition. Together with the Bax/Bcl2 ratio, it could be a good predictive algorithm concerning the isolated cells’ proliferative activity and senescence potential.

Future research should explore strategies to enhance the regenerative potential of aged ASCs, including using small-molecule modulators, epigenetic reprogramming, or exosome-based therapies. Understanding the molecular mechanisms underlying ASCs’ aging will be crucial for optimizing their therapeutic applications and ensuring their safety in regenerative medicine.

## Figures and Tables

**Table 1:** The Spearman correlation coefficient (R) reflects the functional interaction between observed pro-apoptotic (Bax), anti-apoptotic (Bcl2), and proliferative/tumorigenic (CA9 and Oct4) factors in different age groups. Only the significant (*p* < 0.05) correlation coefficients were presented.

Up to 5 Years	Bax	Bcl2	CD 44
CA9	-	0.60	-
**Up to 15 years**			
Bcl2	0.80	-	0.56
Bax	-	-	0.78
CA9	0.68	0.86	0.64
Oct 4	0.62	-	-
CD 90	-	-	0.62
**Over 15 years**			
Bcl2	0.52	-	-
CD 90	0.75	-	0.61

## Data Availability

The data presented in this study are available upon request from the corresponding author.

## Supplementary Materials

The following supporting information can be downloaded at https://www.mdpi.com/article/10.3390/ani15131910/s1, Table S1: Gene abbreviations, full names, forward and reverse primer sequences, qPCR product size, and gene accession numbers [65,66,67]; Table S2: Comparison of the best approximation of equine ASCs from three aged groups at different passages by exponential, logistic, and Gompertzian laws. The estimation of the error has performed using the mean absolute error (MAE) method and the root mean square error (RMSE) method.

## Abbreviations

The following abbreviations are used in this manuscript:

18S	18S ribosomal RNA
ALP	Alkaline phosphatase
ASCs	Adipose-derived mesenchymal stem cells
B2M	β2 microglobulin
Bax	Bcl-2 associated X protein
Bcl-2	B-cell lymphoma 2
CXCR4	C-X-C motif chemokine receptor 4
DMEM	Dulbecco’s Modified Eagle’s Medium
DNMT1	DNA (cytosine-5)-methyltransferase 1
EVs	Extracellular vesicles
FBS	Fetal bovine serum
GAPDH	Glyceraldehyde-3-phosphate dehydrogenase
HKs	Housekeeping genes
HPRT	Hypoxanthine Phosphoribosyl transferase
HS	Heat-inactivated horse serum
IBMX	3-isobutyl-1-methylxanthine
IFATS	International Federation for Adipose Therapeutics and Science
iPSC	Induced pluripotent stem cells
ISCT	International Society for Cellular Therapy
ITS	Insulin-Transferrin-Selenium
LD	lipid droplet
MAE	mean absolute error
MHCII	Major histocompatibility complex class II
MOMP	mitochondrial outer membrane permeabilization
MSCs	Mesenchymal stem cells
PDT	Population doubling time
PSCs	Pluripotent stem cells
RMSE	root mean square error
SDFT	Superficial digital flexor tendon
SSEA4	Stage-specific embryonic antigen-4
SVF	Stromal vascular fraction
TET-2	Tet methylcytosine dioxygenase 2
TET-3	Tet methylcytosine dioxygenase 3
TGFβ1	transforming growth factor beta 1
TRA-1-60	Tumor rejection antigens-1-60
TRA-1-81	Tumor rejection antigens-1-81
YM	Maximum yield
