# Supplementary material for: Transcriptional Factors Related to Cellular Kinetics, Apoptosis, and Tumorigenicity in Equine Adipose-Derived Mesenchymal Stem Cells (ASCs) Are Influenced by the Age of the Donors
Source: Animals (Basel). 2025 Jun 28;15(13):1910. doi: 10.3390/ani15131910 (PMC12249217; doi:10.3390/ani15131910)
Supplement: Supplementary file 1 [file animals-15-01910-s001.zip › animals-3574828-supplementary.pdf]

Table S1. Gene abbreviation, full name, forward and reverse primer sequences, qPCR product size, and gene accession numbers.

A: Housekeepers

Abbreviation	Full name	Forward	Reverse	Product length	NCBI Assecion №
**HPRT** [31]	Hypoxanthine phosphoribosyltransferase	ccagtcaacaggg gacataa	gcttgcgaccttga ccatct	**163**	AY372182.1
**GAPDH** [65]	Glyceraldehyde 3-phosphate dehydrogenase	tccctgcttctactg gtgct	cgtatttggcagctt tctcc	**147**	NM_0011638 56.1
**18S** [32]	18S ribosomal RNA (RN18S)	atgcggcggcgtta ttcc	gctatcaatctgtca atcctgtcc	**204**	NR_046271.1
**B2M** [31]	Beta 2-microglobulin	tttactcacgtcacc cagca	tccacaccattggg agtaaag	**199**	X69083.1

B: CD markers

Abbreviation	Full name	Forward	Reverse	Product length	NCBI Assecion №
**CD29**	Integrin subunit beta 1 (ITGB1)	gtcttggaaccgatct gatg	gagcttggctggtg ttgtac	**119**	NM_0013012 17.1
**CD44** [66]	CD44 molecule	atcctcacgtccaaca cctc	ctcgcctttcttggt gtagc	**165**	NM_0010854 35.2
**CD73** [32]	ecto-5′-nucleotidase	gaagaaggcctttga gcacag	caaagaacatctaa tttgaccac	**134**	XM_0236507 58.1
**CD90** [31]	CD90 Thy-1 cell surface antigen (THY1)	actgagctctcggcac cat	gtgtggcggtggta ttctca	**156**	XM_0015032 25.4
**CD105** [32]	Endoglin	ccgccgcactgtggt acatctac	tgtggttggtgctac tgctctctg	**108**	XM_0033641 44.4
**CD14** [31]	lipopolysaccharide receptor (CD14)	ctgtgaggtggacgat gaga	taggtcctccagcg tcagtt	**301**	AF200416.2
**CD45** [66]	leukocyte common antigen	tgattcccagaaatga ccatgta	acattttgggcttgt cctgtaac	**101**	AY114350.1
**MHC-II** [67]	major histocompatibility complex class II	agcggcgagttgaac ctacagt	cggatcagacctgt ggagatga	**172**	NM_0011428 16

C: Tumorigenic and apoptosis-related markers

Abbreviation	Full name	Forward	Reverse	Product length	NCBI Assecion №
**BCL2**	B-cell lymphoma 2	ggattgtggccttcttt gag	gccggttcaggtat tcagtc	**114**	XM_0014904 36.3
**BAX**	Bcl-2-like protein 4	aggatgcgtccacca agaag	tgccactcggaaaa agacc	**134**	XM_0055967 28.1
**CA9** [32]	Carbonic anhydrase 9 Tumor cell marker	tgcaactgctgctgtta ctg	tcttccccagatga atctcc	**130**	XM_0147358 92.2
**Oct 4**	Octamer-binding transcription factor 4	tggtacgagtgtggtt ctgc	gtgccaggggaaa ggatacc	**129**	XM_0236242 32.1

Table S2. Comparison of the best approximation of equine ASCs from three aged groups at different passages by exponential, logistic, and Gompertzian laws. The estimation of the error has done using the mean absolute error (MAE) method and the root mean square error (RMSE) method.

Horses up to 5 years, passage 3

Function	Min MAE	Parameters	Min RMSE	Parameters
**Exponential**	4.42	C=1.75	5.43	C=1.78
**Logistic**	0.76	K=53.1; r=0.76	1.16	K=52.8; r=0.76
**Gompertz**	0.67	K=170.5; a=0.18	0.92	K=152.1; a=0.19

Horses up to 15 years, passage 3

Function	Min MAE	Parameters	Min RMSE	Parameters
**Exponential**	3.39	C=1.77	4.31	C=1.79
**Logistic**	1.11	K=67.0; r=0.72	1.69	K=73.9; r=0.70
**Gompertz**	1.10	K=426.8; a=0.14	1.72	K=436.3; a=0.14

Horses over to 15 years, passage 3

Function	Min MAE	Parameters	Min RMSE	Parameters
**Exponential**	1.22	C=1.38	1.51	C=1.4
**Logistic**	0.31	K=6.0; r=0.76	0.38	K=5.8; r=0.83
**Gompertz**	0.29	K=6.8; a=0.4	0.40	K=6.4; a=0.43

Horses up to 5 years, passage 4

Function	Min MAE	Parameters	Min RMSE	Parameters
**Exponential**	3.88	C=1.7	4.62	C=1.7
**Logistic**	0.75	K=32.9; r=0.76	0.99	K=33.5; r=0.76
**Gompertz**	0.45	K=61.5; a=0.23	0.97	K=64.5; a=0.23

Horses up to 15 years, passage 4

Function	Min MAE	Parameters	Min RMSE	Parameters
**Exponential**	2.55	C=1.7	3.01	C=1.66
**Logistic**	0.48	K=32.1; r=0.69	0.65	K=31.6; r=0.69
**Gompertz**	0.34	K=77.2; a=0.19	0.44	K=88.5; a=0.18

Horses over to 15 years, passage 4

Function	Min MAE	Parameters	Min RMSE	Parameters
**Exponential**	1.46	C=1.33	1.83	C=1.36
**Logistic**	0.75	K=4.3; r=0.72	0.94	K=4.9; r=0.88
**Gompertz**	0.72	K=4.2; a=0.62	0.95	K=5.0; a=0.54

Horses up to 5 years, passage 5

Function	Min MAE	Parameters	Min RMSE	Parameters
**Exponential**	2.81	C=1.7	3.31	C=1.68
**Logistic**	0.99	K=26.8; r=0.76	1.15	K=33.8; r=0.70
**Gompertz**	0.50	K=98.8; a=0.18	0.60	K=85.2; a=0.19

Horses up to 15 years, passage 5

Function	Min MAE	Parameters	Min RMSE	Parameters
**Exponential**	1.94	C=1.7	2.68	C=1.71
**Logistic**	1.57	K=82.5; r=0.60	2.09	K=63.2; r=0.62
**Gompertz**	1.26	K=219.5; a=0.15	1.76	K=246.1; a=0.14

Horses over to 15 years, passage 5

Function	Min MAE	Parameters	Min RMSE	Parameters
**Exponential**	0.89	C=1.41	1.05	C=1.4
**Logistic**	0.31	K=6.2; r=0.68	0.40	K=6.1; r=0.67
**Gompertz**	0.20	K=7.5; a=0.32	0.28	K=7.5; a=0.31
